# 

**Published:** 1903-12-15

**Authors:** 


### ANNOUNCEMENTS.

The dental profession of Philadelphia, represented by all of its organizations, will celebrate on February 27, 1904, the fiftieth anniversary of the graduation of the Class of 1854 of the Philadelphia College of Dental Surgery by a complimentary banquet to the surviving members of the class, consisting of Drs. Louis Jack, James Truman, C. Newlin Peirce and W. Storer How.

All dentists in good standing are invited to participate. The subscription price, including a banquet ticket and one copy of the souvenir historical volume to be published in commemoration of the event, has been fixed at ten dollars. The subscription list will be open until February 10, 1904.

The committee in charge of the celebration consists of the following members:

Edwin T. Darby, Edward C. Kirk, R. H. D. Swing, Albert N. Gaylord, Earl C. Rice, I. N. Broomell, J. T. Lippincott L. Foster Jack, G. L. S. Jameson, J. D. Thomas, Wilbur F. Litch, H. C. Register, Wm. H. Trueman, Robert Huey, Wm. L. J. Griffin, J. Clarence Salvas, D. N. McQuillen.

Applications together with the subscription may be forwarded to the chairman of the Invitation Committee,

ROBERT HUEY, D.D.S.,
330 S. Fifteenth St., Philadelphia.

FOURTH INTERNATIONAL DENTAL CONGRESS, ST. LOUIS, MO., AUGUST 19 TO SEPTEMBER 3, 1904. The Committee on State and Local Organizations is a committee appointed by the Committee of Organization of the Fourth International Congress with the object of promoting the interests of the Congress in the several States of the Union Each member of the committee is charged with the duty of receiving applications for membership in the Congress under the rules governing membership, as prescribed by the Committee on Membership and approved by the Committee of Organization. These rules provide that *membership in the Congress shall be open to all reputable legally-qualified practitioners of dentistry.* Membership in a State or local society is not a necessary qualification for membership in the Congress.

Each state chairman, as named below, is furnished with official application blanks and is authorized to accept the membership fee of ten dollars from all eligible applicants within his State. The state chairman will at once forward the fee and official application with his indorsement to the chairman of the Finance Committee, who will issue the official certificate conferring membership in the Congress. No application from any of the States will be accepted by the chairman of the Finance Committee unless approved by the state chairman, whose indorsement is a certification of eligibility under the membership rules.

A certificate of membership in the Congress will entitle the holder thereof to all the rights and privileges of the Congress, the right of debate, and of voting on all questions which the Congress will be called upon to decide. It will also entitle the member to one copy of the official transactions when published and to participation in all the events for social entertainment which will be officially provided at the time of the Congress.

The attention of all reputable legally-qualified practitioners of dentistry is called to the foregoing plan authorized by the Committee of Organization for securing membership in the Congress, and the Committee earnestly appeals to each eligible practitioner in the United States who is interested in the success of this great international meeting to make application at once through his state chairman for a membership certificate. By acting promptly in this matter the purpose of the Committee to make the Fourth International Dental Congress the largest and most successful meeting of dentists ever held will be realized, and the Congress will thus be placed upon a sound financial basis.

Let every one make it his individual business to help at least to the extent of enrolling himself as a member and the success of the undertaking will be quickly assured. Apply at once to your state chairman. The state chairmen already appointed are as follows:

*General Chairman*—J. A. Libbey, 524 Penn Av., Pittsburg, Pa.

*States*—Alabama, H. Clay Hassell, Tuscaloosa; Arkansas, W. H. Buckley, 510½ Main St., Little Rock; California, H. P. Carlton, Crocker Bldg., San Francisco; Colorado, H. A. Fynn, Denver; Connecticut, Henry McManus, 92 Pratt St., Hartford; Delaware, C. R. Jeffries, New Century Bldg., Wilmington; District of Columbia, W. N. Cogan, The Sherman, Washington; Florida, W. G. Mason, Tampa; Georgia, H. H. Johnson, Macon; Idaho, J. B. Burns, Payette; Indiana, H. C. Kahlo, 115 E. New York St., Indianapolis.; Iowa, W. R, Clack, Clear Lake; Kansas, G. A. Esterly, Lawrence; Kentucky, H. B. Tileston, 314 Equitable Bldg., Louisville; Louisiana, Jules J. Sarrazin, 108 Bourbon St., New Orleans; Maryland, W. G. Foster, 813 Eutaw St., Baltimore; Massachusetts, M. C. Smith, 3 Lee Hall, Lynn; Michigan, G. S. Shattuck, 539 Fourth Av., Detroit; Minnesota, C. A. Van Duzee, 51 Germania Bank Bldg., St. Paul; Missouri, J. W. Hull, Altman Bldg., Kansas City; Nebraska, H. A. Shannon, 1136 "O" St., Linco'n; New Jersey, Alphonso Irwin, 425 Cooper St., Camden; New York, B. C. Nash, 142 W. 78th St., New York City; North Carolina, C. L. Alexander, Charlotte; Ohio, Henry Barnes, 1415 New England Bldg., Cleveland; Oklahoma, T. P. Bringhurst, Shawnee; Pennsylvania, H. E. Roberts, 1516 Locust St., Philadelphia; Rhode Island, D. F. Keefe, 315 Butler Exchange, Providence; South Carolina, J. T. Calvert, Spartanburg; Tennessee, J. P. Gray, Berry Block, Nashville; Texas, J. G. Fife, Dallas; Utah, W. L. Ellerbeck, 21 Hooper Bldg., Salt Lake City; Virginia, F. W. Stiff, Richmond; West Virginia, H. H. Harrison, 1141 Main St., Wheeling; Wisconsin, A. D. Gropper, 401 E. Water St., Milwaukee.

For the Committee of Organization,

EDWARD C. KIRK, *Secretary*.

